# Pathogenicity of egg-type duck-origin isolate of Tembusu virus in Pekin ducklings

**DOI:** 10.1186/s12917-019-2136-x

**Published:** 2019-10-24

**Authors:** Te Liang, Xiaoxiao Liu, Shenghua Qu, Junfeng Lv, Lixin Yang, Dabing Zhang

**Affiliations:** 1 Key Laboratory of Animal Epidemiology of the Ministry of Agriculture, College of Veterinary Medicine, China Agricultural University, No. 2 Yuanmingyuan West Road, Haidian district, Beijing, 100193 People’s Republic of China; 2 State Key Laboratory of Proteomics, Beijing Proteome Research Center, National Center for Protein Sciences-Beijing (PHOENIX Center), Beijing Institute of Lifeomics, Beijing, China

**Keywords:** Duck, Tembusu virus, TMUV-caused disease, Pathogenicity

## Abstract

**Background:**

Tembusu virus (TMUV) usually affects adult ducks, causing a severe drop of egg production. It has also been shown to be pathogenic in commercial Pekin ducklings below 7 weeks of age. Here, we report a TMUV-caused neurological disease in young egg-type ducklings and the pathogenicity of the egg-type duck-origin TMUV isolates in meat-type Pekin ducklings.

**Results:**

The disease occurred in 25 to 40-day-old Jinding ducklings in China, and was characterized by paralysis. Gross lesions were lacking and microscopic lesions appeared chiefly in brain and spleen. Inoculation in embryonated duck eggs resulted in isolation of TMUV Y and GL. The clinical signs and microscopic lesions observed in the spontaneously infected egg-type ducks were repeated in Pekin ducklings by experimental infection. Notably, both Y and GL strains caused 100% mortality in the case of 2-day-old inoculation by intracerebral route. High mortalities (80 and 70%) also occurred following infection of the Y virus at 2 days of age by intramuscular route and at 9 days of age by intracerebral route.

**Conclusions:**

These findings demonstrate that the egg-type duck-origin TMUVs exhibit high pathogenicity in Pekin ducklings, and that the severity of the disease in ducklings is dependent on the infection route and the age of birds at the time of infection. The availability of the highly pathogenic TMUV strains provides a useful material with which to begin investigations into the molecular basis of TMUV pathogenicity in ducks.

## Background

Tembusu virus (TMUV) is small, enveloped virus that contains a single-stranded positive-sense RNA genome approximately 11 kb in length [1]. Based on phylogenetic analysis, TMUV is classified within the genus *Flavivirus* in the family *Flaviviridae* [2, 3]. On the basis of mode of transmission and serological cross-reactivity, the virus is also classified as belonging to Ntaya virus (NTAV) serocomplex in mosquito-borne group [4]. TMUV was originally isolated from mosquitoes of the genus *Culex* in Malaysia in 1955. Since then, TMUV has been known to cause infection in chicks [5, 6], geese [1, 7], sparrows [8], pigeons [9], and humans [10–12]. There is already evidence suggesting that TMUV can be transmitted by various routes, including mosquito-borne [13], airborne, direct contact [14], and vertical transmission [15]. Wild birds may also have played a role in spread of the disease [8].

TMUV-caused disease was first reported in 2010 in China [2, 16, 17]. Subsequently, the disease was documented in Malaysia and Thailand [18, 19]. In outbreaks, TMUV infection most commonly affects adult breeder and layer ducks. Affected ducks display specific clinical signs, characterized by dramatic drop in feed take and egg production. Onset and spread of the disease are very rapid. Practically all clinical signs in a flock occur within 7–10 days. Gross pathological changes appear chiefly in the ovary, which is degenerate and exhibits hemorrhages [2, 16, 17]. Previous works have also demonstrated that TMUV is pathogenic in young ducklings. The first reported outbreaks of spontaneous TMUV-related neurological disease were observed in 20-day-old Pekin ducklings (*Anas platyrhynchos domesticus*) in China [20]. According to a previously published description, a TMUV-related disease in ducklings, known locally as “duck leg paralysis/lameness”, has been circulated in Malaysia for several years. The disease resulted in losses of up to 25 and 29% in 4 to 7-week-old broiler Pekin ducks (strain Cherry Valley) due to culling or perishing of seriously affected birds [18]. The pathogenicity of TMUV in ducklings below 7 weeks of age has been confirmed by experimental infections [18, 20–23].

The TMUV-related disease can be reproduced by experimental infections via multiple routes of infection, such as oral administration, nasal drip, and subcutaneous, intramuscular, intracerebral and intravenous injections [18, 20–23]. In the study by Yun et al. [20], experimental infections of 1-day-old Pekin ducklings were conducted by three different routes (intracerebral, subcutaneous, and intranasal), which showed that mortality (20%) was only caused by intracerebral inoculation. This investigation indicates that the routes of infection may play an important role in experimental infection. Further studies by Li et al. [21], Lu et al. [22], and Sun et al. [23], which showed that mortality (18 and 30%) was caused in 5 to 7-day-old Pekin ducklings (*Anas platyrhynchos domesticus*; strain Cherry Valley) following infection by the intramuscular and intranasal routes, but no mortality occurred following infection at more than 2 weeks of age, supported the view that the severity of the disease may be influenced by the age of the birds at the time of infection. Sun et al. (2014) suggested that the age-related differences in the resistance to TMUV infection should be considered in investigation of the TMUV pathogenicity in ducks [23].

Experimental infections of day-old chickens demonstrated that TMUV isolates may vary with regard to pathogenicity [5, 13]. In the study by Kono et al. (2000) [5], the TMUV Sitiawan isolate, recovered from broiler chickens in Malaysia in 2000, was shown to cause clinical signs in a low proportion of specific pathogen free (SPF) chickens in the case of 1-day-old infection. However, the Thai-MLO305 TMUV, which was isolated from mosquitoes collected in 2002 in Thailand, was found to cause high mortality (50 and 100%) of leghorn chickens following infection at 1 day of age and less than 1 day of age [13].

In 2014, a disease characterized by paralysis occurred in Northeast China in three flocks of 25 to 40-old-day egg-type ducks (*Anas platyrhynchos domesticus*), locally known as Jinding ducks. The aim of the present study was to isolate the causative agent of the disease and to investigate its pathogenicity in young ducklings using a Pekin duckling infection model.

## Results

### Clinical features of the disease in young Jinding ducks

**Fig. 1:**
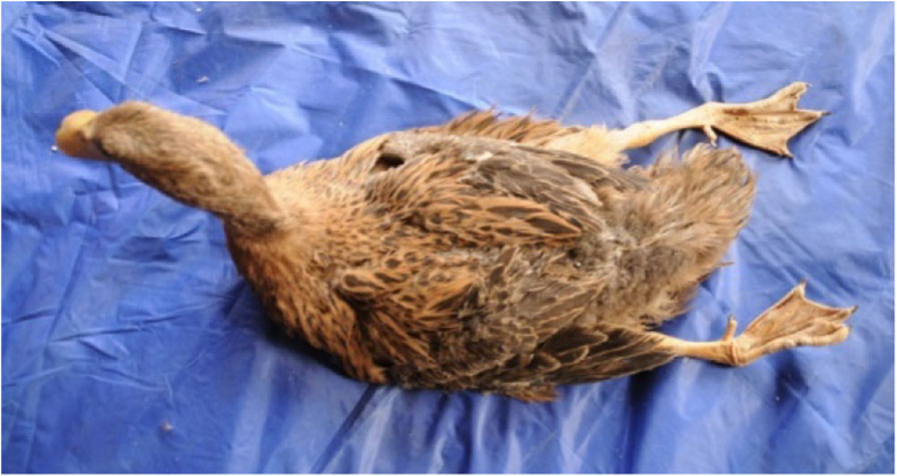
74-day-old Jinding duck spontaneously infected with TMUV

In January 2014, we were contacted to conduct an epidemiological investigation in Northeast China due to the occurrence of a disease in a flock (designated A) of Jinding ducks aged 74 days. Clinical observation revealed that principle signs of affected ducks were leg paralysis (Fig. 1). The flock owner reported that the disease, characterized by paralysis and drop in feed intake, was noted at 40 days of age. Of 7000 birds in the flock, about 2100 (30%) were paralysed at the first week after onset of the disease. At the peak of paralysis, the feed intake decreased by 60–70%. At the visit, most of affected ducks have recovered from their illness. Mortality was negligible.

In October 2014, 10 clinically ill Jinding ducks from two flocks (designated B and C) of two farms situated in Northeast China were sent to our laboratory for the purpose of diagnosis. The cases included five 32-day-old birds from flock B and five 34-day-old birds from flock C. In flocks B and C, clinical signs appeared at 25 and 27 days of age respectively. Of 3000 birds in flock B and 3200 birds in flock C, about 10 and 15% showed signs of paralysis, respectively. A drop in feed intake was also seen in the two flocks. Mortality was low, ranging from 3 to 5%.

### Gross and microscopic lesions of the affected Jinding ducks

**Fig. 2:**
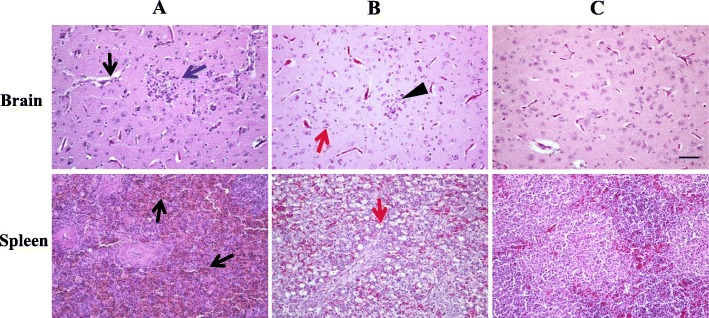
Microscopic lesions. Penal **a**, 34-day-old clinically ill Jinding ducks; penal **b**, Pekin ducklings died from infection with the TMUV GL isolate; penal **c**, uninfected Pekin duck control. Lesions observed in each tissue were as follows: Brain, edema (black arrow), local glial cell proliferation (blue arrow), necrosis of neuron (red arrow), and increase of microglia (triangle); Spleen, hemorrhages (black arrow) and lymphocyte necrosis and emptying (red arrow). Bar = 50 μm

At necropsy, no obvious gross pathological changes were observed in internal organs of affected Jinding ducks. Whereas histopathological examination of tissues collected from five 34-day-old ducks in flock C revealed that the presence of microscopic lesions (Fig. 2; Table 1). The principal microscopic lesions appeared to be multifocal gliosis in the brain (4/5), with moderate degree of severity. Very mild edema was seen in the brain of all ducks. Occasionally, mild cerebral congestion was also observed. Most cases (4/5) had lesions of mild hemorrhages in the spleen. Other microscopic changes were found in partial cases, such as lymphocyte necrosis and evacuation in spleen, hepatocyte steatosis and lymphocetic infiltration in liver, renal tubulointerstitial congestion and lymphocytic infiltration in kidney, inflammatory cell infiltration in heart, and acinar cell necrosis and lymphocytic infiltration in pancreas. The severities of these lesions were all mild.

**Table 1:** Microscopic lesions in tissues of ducks spontaneously and experimentally infected by TMUV^a^

Tissue	Microscopic lesion	Spontaneously infected Jinding ducks	Experimentally infected Pekin ducks
Brain	Edema	+ (5)	+++ (5)
	Multifocal gliosis	+++ (4)	+++ (3)
	Congestion	++ (1)	++++ (2)
	Neuronal swelling	–	++ (3)
	Neuronal necrosis	–	++ (1)
	Lymphocytic infiltration	–	+++ (1)
Spleen	Hemorrhage	++ (4)	–
	Lymphocyte necrosis and evacuation	++ (2)	++++ (5)
Liver	Hepatocyte steatosis	+ (3)	++ (1)
	Lymphocytic infiltration	+ (3)	++ (5)
	Congestion	+ (1)	–
	Hepatic sinusoid dilatation	–	++ (5)
Kidney	Renal tubulointerstitial congestion	+ (2)	+ (1)
	Lymphocytic infiltration	++ (2)	–
	Renal tubular epithelial cell necrosis	–	+ (1)
Heart	Inflammatory cell infiltration	++ (2)	ND
Pancreas	Acinar cell necrosis	+ (3)	ND
	Lymphocytic infiltration	++ (2)	ND

^a^ – = no change, + = very mild; ++ = mild; +++ = moderate; ++++ = marked. Five Jinding ducks collected from flock C and five ducks died from infection with the TMUV GL isolate were used for histopathological examination. Data in parentheses indicates no. of ducks with the severities of microscopic lesions as indicated. ND, not detected

### Identification of etiological agent of the duck disease

Our results indicated that the affected ducks in flocks A, B, and C exhibited signs typical of TMUV infection in commercial Pekin ducks [18]. To identify the etiological agent of the disease, a reverse transcription (RT)-PCR assay based on previously reported primers [24] was used to attempt to amplify TMUV-specific cDNA fragments from RNAs extracted from tissues of ducks taken from flocks A, B, and C. The two ducks in flock A, from which liver samples were collected, and all the ten ducks in flocks B and C, from which multiple tissues were collected, were tested positive for TMUV.

To understand tissue distribution of TMUV in spontaneously infected ducks, the RT-PCR was performed to test tissues collected from the ten ducks in flocks B and C. TMUV RNA was most frequently detected in heart (10/10), followed by spleen (9/10), brain (8/10), kidney (6/10), pancreas (5/10), and liver (3/10). In one duck TMUV RNA was detected in all tissues tested, and in nine other ducks two to five tissues was positive.

**Fig. 3:**
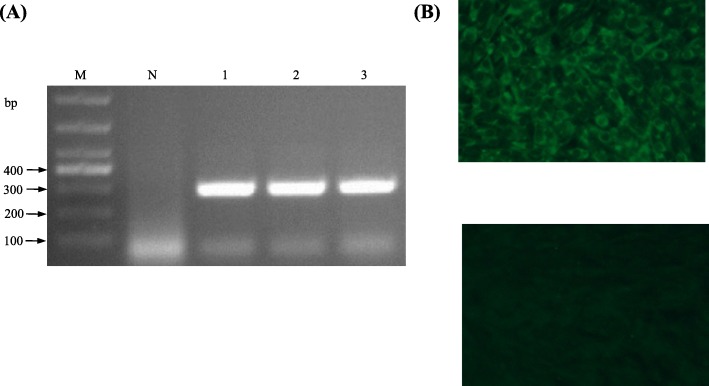
Characterization of TMUV isolates. **a** Detection of TMUV Y by RT-PCR. Lane M, molecular weight marker; lane N, negative control; lanes 1–3 indicate the third to fifth passages of Y virus respectively. **b** Identification of TMUV GL by IFA. Top, BHK-21 cells infected with TMUV GL; bottom, negative control

Inoculation and repeated passages in 8 to 9-day-old embryonated Pekin duck eggs resulted in isolation of two TMUV strains, including strain Y from a liver sample from flock A and strain GL from a kidney sample from flock C. The clarified liver and kidney suspensions caused deaths of Pekin duck embryos at 4 days post inoculation (PI). In the subsequent passages all infected embryos died within 2–4 days PI. Dead embryos exhibited severe subcutaneous hemorrhages. The Y and GL viruses were passaged four and two times in embryonated duck eggs, respectively. TMUV was detectable in all inoculated embryos (Fig. 3a), which was confirmed by nucleotide sequence determination and analysis of the amplified fragments. No amplification occurred when nucleic acids extracted from the Y and GL isolates were subjected to PCR-based assays specific to other viruses including avian influenza virus (AIV), Newcastle disease virus (NDV), duck astrovirus (DAstV), duck enteritis virus (DEV), and duck hepatitis A virus (DHAV). Allantoic fluids collected from Y- and GL-infected embryos did not agglutinate erythrocytes of chickens.

The Y and GL viruses were characterized further by cultivation in BHK cells and by using indirect immunofluorescence (IIF) assay. Inoculation of the Y and GL isolates onto confluent monolayers of BHK-21 cells resulted in the development of cytopathic effect (CPE) at 72–96 h PI. In the immunofluorescence test performed with chicken antiserum against TMUV and fluorescein isothiocyanate (FITC)-conjugated goat anti-chicken IgG, the Y- and GL-infected cells showed fluorescence (Fig. 3b).

To understand the molecular features of the egg-type duck-origin isolates, we determined the full-length genome sequences of the Y and GL viruses. Both Y and GL genomes consisted of 10,990 nucleotides and had a typical TMUV organization. The genome sequence of isolate Y was found to be 100% identical to that recently determined by other researchers in our laboratory (GenBank accession no. MK542820). Pairwise comparisons of the genome and polyprotein sequences of the Y and GL isolates with previously reported TMUV isolates (e.g., isolates YY5 and ZJ-6) [1, 2, 20] showed that they shared high identity at the nucleotide (genome: 98–99%) and amino acid (polyprotein: 99%) with each other. Detailed examination of the polyprotein sequences of the Y and GL isolates revealed the presence of three amino acid changes.

### Pathogenicity of the Y and GL isolates in Pekin ducklings

**Fig. 4:**
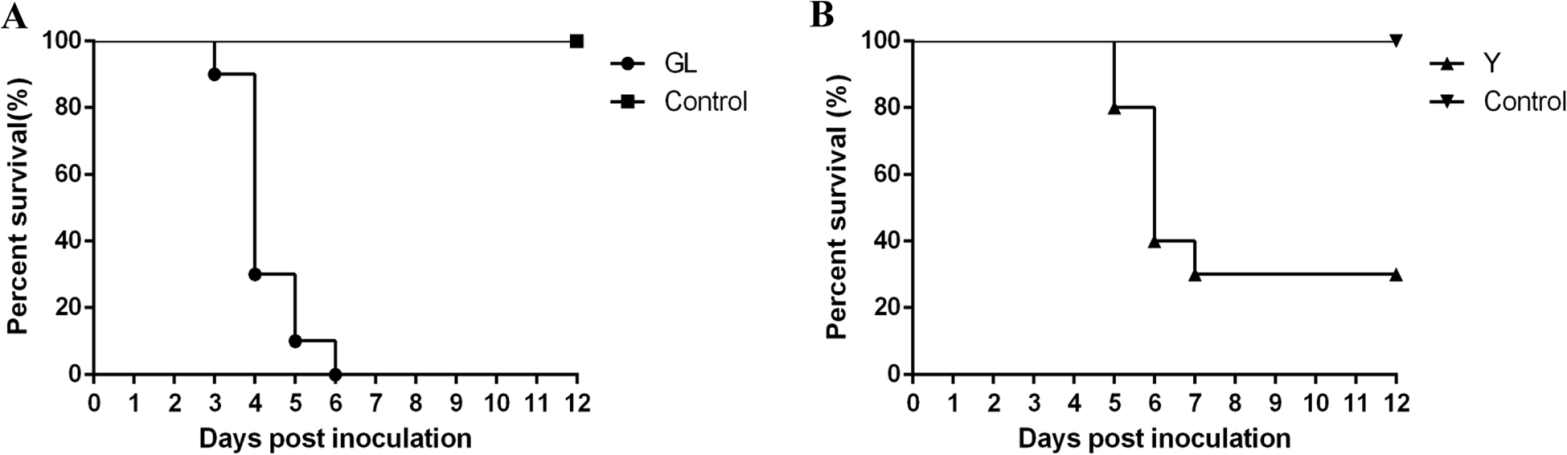
Survival curves of Pekin ducklings infected with TMUV. The results shown are representative of two independent experiments, in which ducklings were infected intracerebrally with the GL (**a**) and Y (**b**) isolates of TMUV at 2 and 9 days of age, respectively

The pathogenicity of strain Y in 2-day-old Pekin ducklings was first evaluated by intramuscular and intracerebral inoculation, respectively. Infection of ducklings resulted in the appearance of clinical signs, including severe drop of feed intake, depression, listlessness, head tremors, ataxia, and difficulty in walking. Intramuscular inoculation resulted in 80% mortality, which occurred within 5–14 days. In cases of intracerebral inoculation, all ducklings died within 3–5 days PI. Subsequently, the pathogenicity of strain GL in 2-day-old Pekin ducklings was investigated by intracerebral inoculation. The virus caused clinical signs similar to those caused by strain Y, and 100% mortality within 3–6 days PI (Fig. 4a).

The pathogenicity of TMUV Y in Pekin ducklings was further investigated by intracerebral inoculation at 9 days of age. At 3 days PI, ducklings exhibited clinical signs indicative of TMUV infection, e.g., severe drop in feed intake and neurological signs characterized by tremor, ataxia, and difficulty in walking. A 70% mortality resulted, which occurred within 5–7 days PI (Fig. 4b).

At necropsy, no specific gross lesions were found in the organs of ducklings dead from infection with strains Y and GL. Occasionally an enlarged spleen was seen. Histopathological examination of tissues (brain, liver, spleen, and kidney) from dead ducklings in the second experiment revealed that the microscopic lesions appeared chiefly in the spleen and brain (Table 1, Fig. 2). The most marked and consistent microscopic lesions were splenic lymphocytic necrosis and evacuation. In all ducklings, edema was seen in the brain, which was more serious than that of the natural cases. In most cases (3/5), multifocal gliosis was detected in the brain, with degree of severity comparable to that of the natural cases. A small portion (2/5) of cases had lesions of marked cerebral congestion. Three of five ducks displayed mild neuronal swelling. Occasionally, mild neuronal necrosis and moderate lymphocytic infiltration were seen in the brain. Other microscopic changes, such as mild lymphocytic infiltration and hepatic sinusoid dilatation in liver and renal tubulointerstitial congestion in kidney were also observed. The similar microscopic lesions in the spleen and brain could also been observed from the dead ducklings in the third experiment. No clinical signs, lesions and deaths were observed in uninfected control groups during the whole experiment period. TMUV RNA was detectable in all brain and spleen sampled from dead ducks (*n* = 4) in the third experiment by the RT-PCR assay.

## Discussion

The present paper reports a TMUV-caused neurological disease occurred in young egg-type ducks. We showed that the disease was characterized by clinical signs of paralysis, and microscopic lesions in brain and spleen. Similar results were obtained from experimental infections of 2 and 9-day-old Pekin ducklings. These findings are consistent with previous observations of young meat-type Pekin ducks [18–23] and we consider it likely that a presumptive diagnosis of TMUV infection in young ducks can be made based on characteristic clinical features and histopathological changes of brain (e.g., edema and multifocal gliosis) and spleen (e.g., splenic lymphocyte necrosis and evacuation).

The detection of TMUV RNA in multiple internal organs of spontaneously infected ducks suggested that the virus has a broad tissue tropism. TMUV RNA was most frequently detected in the heart, spleen and brain, suggesting that these tissues may be a major site for viral persistence and/or replication. Use of more sensitive tests (e.g., real-time fluorescence quantitative PCR) may be of help to more precise conclusion regarding the tissue distribution of TMUV in infected ducklings. The inoculation of the clinical samples into embryonated Pekin duck eggs and the propagation of the virus in BHK-21 cells suggested that the egg-type duck-origin viruses have a good adaptation to embryonated Pekin duck eggs and BHK-21 cells.

The pathogenicity of TMUV in ducks was originally reflected by its influence on egg production of adult breeder and egg-laying ducks [2, 16, 17, 19]. Circulation of TMUV in flocks of adult ducks in a wide range provides an opportunity for transmission of the virus to ducklings, including meat-type breeder and egg-type ducks during the brood stage and commercial meat-type ducklings. In the present study, we focused on the pathogenicity of the egg-type duck-origin isolates in Pekin ducklings since Pekin duck is the major duck species reared worldwide. We showed that TMUV caused a severe disease with high mortality when infection was performed at 2 and 9 days of age. Our data, in conjunction with previous evidence [18–23], supports the view that young ducklings can be used as the animal model for the study of pathogenicity of TMUV isolates and immunogenicity of TMUV vaccine. It should also be noted that although the TMUV-related disease occurred less frequently in ducklings than in adult ducks, the high pathogenicity of TMUV in ducklings described in this study has raised a concern about the threat to commercial duck farms and the effect on duck meat production.

In the initial investigation of pathogenicity of strain Y in 2-day-old Pekin ducklings, mortality caused by intracerebral inoculation (100%) was higher than that by intramuscular inoculation (80%), demonstrating that the severity of the disease in ducklings is dependent on the route of infection. Observation of strain GL confirmed further that TMUV exhibits high pathogenicity in Pekin ducklings when infection was performed at 2 days of age via intracerebral route. Birds infected at an older age (9-day-old) showed a lower mortality (70%), indicating that the severity of the disease in ducklings also depends on the age of the birds at the time of infection. Our data, together with previous reports [18, 20–23], supports the view that inoculation into the brain appears to be the most sensitive route for evaluating the pathogenicity of the virus. In Pekin ducklings both Y and GL isolates caused significantly higher (70–100%) mortality when compared with those documented in the literature [18–23], suggesting that the severity of the disease occurring in ducklings may also be related to virulence of virus strains. Together, these findings indicate that the severity of diseases caused by virus infection depends on multiple factors. We have noted that the Y and GL isolates share high levels of sequence identity with previously reported TMUVs isolated from adult ducks. We consider it likely that mutation(s) occurred in a few positions in the TMUV genome might alter virulence of the virus in ducks. Other factors may also be involved in pathogenicity of TMUV in ducklings, such as dosage of virus inoculation and level of maternal antibody in ducklings. Previous works have shown that TMUV can be transmitted by multiple routes [8, 13–15], which may also result in disparate pathogenic outcomes. Further studies are needed for clarification of TMUV pathogenesis.

## Conclusions

Taken together, we have demonstrated that TMUV can cause a neurological disease in young egg-type ducklings, characterized by paralysis and microscopic lesions appearing chiefly in brain and spleen. The virus has a broad tissue tropism in egg-type ducklings, and can be isolated by inoculation of tissue samples into embryonated Pekin duck eggs. Laboratory infection experiments demonstrated that the egg-type duck-origin TMUVs exhibit high pathogenicity in Pekin ducklings, and that the severity of the disease in ducklings is dependent on the infection route and the age of birds at the time of infection. More importantly, the severity of the disease in ducklings may be related to virulence of virus strains. The availability of the highly pathogenic TMUV strains provides a useful material with which to begin investigations into the molecular basis of TMUV pathogenicity in ducks.

## Methods

### Sample collection

Samples were taken from three flocks of Jinding ducks in Northeast China in 2014. Ten ducks in flock A were examined and liver samples were collected from two ducks. Six different tissues (heart, liver, brain, spleen, kidney, and pancreas) were sampled from all ducks collected from other two flocks, including five 32-day-old ducks from flock B and five 34-day-old ducks from flock C.

### Sample processing and extraction of RNA

The samples were processed as 20% suspensions with normal saline as described previously [25]. The suspensions were clarified by centrifugation and filtration. RNA was extracted from 250 μl of filtrate using a TRIzol Reagent (Transgen, Beijing, China), and eluted in 40 μl RNase-Free water.

### RT-PCR

A RT-PCR method, employing two pairs of primers reported previously [24], was used to attempt to detect TMUV RNA. 10 μl of RNA was mixed with 4 μl of reverse primer (10 pmol/μl), incubated at 70 °C for 5 min, and then chilled on ice. Subsequently, 10 μl of 5 × MLV buffer, 10 μl of dNTP (10 mM each) (Vazyme, Nanjing, China), 2 μl of M-MLV (Promega, Madison, WI, USA), 1 μl of RNase inhibitor (TaKaRa, Dalian, China), and DEPC-treated H_2_O were added in a final volume of 50 μl. The RT step was carried out at 42 °C for 1 h, followed by incubation at 94 °C for 5 min. 5 μl of cDNA was used for PCR amplification. The reaction mixture contained 12.5 μl of 2 × Taq Master Mix (Vazyme, Nanjing, China), 1 μl (10 pmol/μl) of each of the primers, and 5.5 μl of ddH_2_O. PCR was performed using protocols as described previously [24]. PCR products were analyzed by using 1.5% agarose gel electrophoresis.

### Virus isolation

For the purpose of diagnosis and pathogenicity analysis, embryonated Pekin duck eggs were used to isolate TMUV from a liver sample collected from flock A and a kidney sample collected from flock C. Virus isolation was performed by inoculation of 0.2 ml of filtrate into the allantoic cavity of 8 to 9-day-old embryonated Pekin duck eggs. Embryos were incubated at 37 °C for 5 days. The allantoic fluids and bodies of embryos died at 2–5 days PI were harvested, homogenized, and clarified as described previously [25]. The resulting filtrate was harvested for additional passages. The viruses isolated from flocks A and C, designated strains Y and GL, were passaged four times and twice in embryonated Pekin duck eggs, respectively. For the two viruses, the filtrate prepared from each passage was tested by using the TMUV RT-PCR assay following the protocol described above. All PCR amplicons with the expected product size (approximately 300 bp) were subjected to DNA sequencing for confirmation. To exclude other frequently occurred duck viruses in the Y and GL isolates, including AIV, NDV, DAstV, DEV, and DHAV, previously reported PCR-based assays and hemagglutination test were performed as described previously [2, 25–27].

### Propagation of the isolates in BHK-21 cells

For the purpose of virus identification, the growth property of the Y and GL isolates in BHK-21 cells were investigated. Confluent monolayers of BHK-21 cells were prepared in T25 flasks in growth medium consisting of Dulbecco’s modified Eagle’s medium (DMEM; Macgene, Beijing, China) supplemented with 10% fetal calf serum (FCS; Gibco, NY, USA), 100 U/ml penicillin, and 0.1 mg/ml streptomycin. The inoculum was prepared in 10-fold dilution of the virus with maintenance medium, which consisted of DMEM supplemented with 2% FCS, 100 U/ml penicillin, and 0.1 mg/ml streptomycin. 1 ml of the diluted virus was inoculated onto the cell monolayer. After adsorption at 37 °C for 1.5 h, 5 ml of maintenance medium was added to each flask. The cells were incubated at 37 °C, and checked daily for CPE. When a noticeable CPE was observed, the cell cultures were subjected to one cycle of freezing–thawing, and clarified by centrifugation. Cell-free supernatants were harvested and passaged twice in BHK-21 cells.

### IIF assay

The Y and GL isolates were further characterized by using IIF assay. Each of confluent monolayers of BHK-21 cells grown on a 24 well plate was inoculated with 0.2 ml of the cell-derived virus diluted in maintenance medium. After 1 h at 37 °C for virus adsorption, 0.5 ml maintenance medium was added, and incubation was continued for an additional 36–60 h. The cells were fixed with cold absolute alcohol at room temperature for 15 min. After alcohol was removed, the cells were washed with phosphate-buffered saline (PBS), and inoculated with 0.2 ml of 25-fold dilution of chicken anti-TMUV serum which was prepared previously in our laboratory. After incubation at 37 °C for 1 h, the wells were washed three times with PBS and stained with 0.2 ml of 100-fold dilution of FITC-conjugated goat anti-chicken IgG (KPL, MD, USA). After a further incubation at 37 °C for 45 min, the wells were washed again with PBS, and examined using a fluorescent microscope (Olympus, Tokyo, Japan).

### Animal experiments

Newly hatched Pekin ducklings were provided by Dr. Shuisheng Hou, institute of Animal Science, Chinese Academy of Agricultural Sciences, Beijing, People’s Republic of China. At day 2, the ducklings were used in three experiments to investigate the pathogenicity of the Y and GL isolates. In the first experiment, ducklings were divided into four groups (5 birds/group): two groups were inoculated intramuscularly and intracerebrally with 1.5 × 10^3.375^ 50% egg lethal dose (ELD_50_) of the Y virus, and the other two groups were inoculated intramuscularly and intracerebrally with equal volume of normal saline, respectively. In the second experiment, ducklings were divided into two groups (10 birds/group): one group was inoculated by intracerebral route with 10^4^ ELD_50_ of the GL isolate, and the other with equal volume of normal saline. The third experiment was designed to investigate the pathogenicity of the Y isolate in 9-day-old ducklings. One group (10 birds/group) of ducklings was inoculated intracerebrally with 1.5 × 10^3.375^ ELD_50_ of the virus, and the other with equal volume of normal saline. In each experiment, challenged ducklings and control group were reared in isolators and monitored daily for 12–14 days. Dead ducklings were examined for gross lesions. At the end of all experiments, the survived ducklings were euthanized by administration of sodium pentobarbital with an intravenous dose of 100 mg/kg body weight as described previously [28]. Brain, liver, spleen, and kidney of 5 dead birds in the second experiment were sampled for histopathological examination. Brain and spleen of 4 dead birds in the third experiment were also collected for histopathological examination and molecular detection by using RT-PCR as described above.

### Histopathology

The tissues were placed in 10% buffered formalin, and fixed at room temperature for 48 h. The fixed tissues were embedded in paraffin and cut into about 5-μm-thick sections. After deparaffinization, the sections were stained with haematoxylin and eosin (H & E). Pathological changes were observed under an Olympus microscope (Olympus, Tokyo, Japan). For each tissue, microscopic lesions were divided into 5-grade severities of no change, very mild, mild, moderate, and marked, respectively (Table 1).

### Sequence determination and analysis

The third passages of the Y and GL viruses in BHK-21 cells were subjected to full-length genome sequencing as described previously [27]. Sequence data were processed and assembled with the SeqMan program (LaserGene System Software v5.0, DNAStar Inc., Madison, WI). ORF prediction and translation of the ORF into amino acid were carried out with DNAMAN 5.2.2 (Lynnon). Pairwise sequence comparison of nucleotide and deduced amino acid sequences were performed with the ClustalW software (https://www.genome.jp/tools-bin/clustalw).

### Nucleotide sequence accession number

The genome sequence of the TMUV GL isolate determined in this study has been deposited in GenBank under the accession number MK889501.

## Data Availability

Most data supporting the findings are provided within the text. Original data are available from the corresponding author on reasonable request. The link of the ClustalW software is https://www.genome.jp/tools-bin/clustalw. The link of the Nucleotide database is https://www.ncbi.nlm.nih.gov/nucleotide/. The nucleotide sequence of the TMUV GL isolate under accession number MK889501 is available online (https://www.ncbi.nlm.nih.gov/Genbank/update.html).

## Abbreviations

AIV: Avian influenza virus
CPE: Cytopathic effect
DAstV: Duck astrovirus
DEV: Duck enteritis virus
DHAV: Duck hepatitis A virus
DMEM: Dulbecco’s modified Eagle’s medium
ELD_50_: 50% egg lethal dose
ELISA: Enzyme-linked immunosorbent assays
FCS: Fetal calf serum
FITC: Fluorescein isothiocyanate
IIF: Indirect immunofluorescence
NDV: Newcastle disease virus
ORF: Open reading frame
PBS: Phosphate-buffered saline
PCR: Polymerase chain reaction
PI: Post inoculation
RT-PCR: Reverse transcription PCR
TMUV: Tembusu virus

## References

[1] Yun T, Zhang D, Ma X, Cao Z, Chen L, Ni Z, Ye W, Yu B, Hua J, Zhang Y, Zhang C (2012). Complete genome sequence of a novel flavivirus, duck Tembusu virus, isolated from ducks and geese in China. J Virol.

[2] Cao Z, Zhang C, Liu Y, Liu Y, Ye W, Han J, Ma G, Zhang D, Xu F, Gao X, Tang Y, Shi S, Wan C, Zhang C, He B, Yang M, Lu X, Huang Y, Diao Y, Ma X, Zhang D (2011). Tembusu virus in ducks, China. Emerg Infect Dis.

[3] Kuno G, Chang GJJ, Tsuchiya KR, Karabatsos N, Cropp CB (1998). Phylogeny of the genus *Flavivirus*. J Virol.

[4] Maeda A, Maeda J (2013). Review of diagnostic plaque reduction neutralization tests for flavivirus infection. Vet J.

[5] Kono Y, Tsukamoto K, Abd HM, Darus A, Lian TC, Sam LS, Yok CN, Di KB, Lim KT, Yamaguchi S, Narita M (2000). Encephalitis and retarded growth of chicks caused by Sitiawan virus, a new isolate belonging to the genus *Flavivirus*. Am J Trop Med Hyg..

[6] Chen S, Wang S, Li Z, Lin F, Cheng X, Zhu X, Wang J, Chen S, Huang M, Zheng M (2014). Isolation and characterization of a Chinese strain of Tembusu virus from Hy-line Brown layers with acute egg-drop syndrome in Fujian, China. Arch Virol.

[7] Huang X, Han K, Zhao D, Liu Y, Zhang J, Niu H, Zhang K, Zhu J, Wu D, Gao L, Li Y (2013). Identification and molecular characterization of a novel flavivirus isolated from geese in China. Res Vet Sci.

[8] Tang Y, Diao Y, Yu C, Gao X, Ju X, Xue C, Liu X, Ge P, Qu J, Zhang D (2013). Characterization of a Tembusu virus isolated from naturally infected house sparrows (*Passer domesticus*) in northern China. Transbound Emerg Dis.

[9] Liu P, Lu H, Li S, Moureau G, Deng YQ, Wang Y, Zhang L, Jiang T, de Lamballerie X, Qin CF, Gould EA, Su J, Gao GF (2012). Genomic and antigenic characterization of the newly emerging Chinese duck egg-drop syndrome flavivirus: genomic comparison with Tembusu and Sitiawan viruses. J Gen Virol..

[10] Smith R, Woodall JP, Whitney E, Deibel R, Gross MA, Smith V, Bast TF (1974). Powassan virus infection. A report of three human cases of encephalitis. Am J Dis Child.

[11] Olson JG, Ksiazek TG, Gubler DJ, Lubis SI, Simanjuntak G, Lee VH, Nalim S, Juslis K, See R (1983). A survey for arboviral antibodies in sera of humans and animals in Lombok, Republic of Indonesia. Ann Trop Med Parasitol.

[12] Tang Y, Gao X, Diao Y, Feng Q, Chen H, Liu X, Ge P, Yu C (2013). Tembusu virus in human, China. Transbound Emerg Dis.

[13] O'Guinn ML, Turell MJ, Kengluecha A, Jaichapor B, Kankaew P, Miller RS, Endy TP, Jones JW, Coleman RE, Lee JS (2013). Field detection of Tembusu virus in western Thailand by RT-PCR and vector competence determination of select *culex* mosquitoes for transmission of the virus. Am J Trop Med Hyg.

[14] Li X, Shi Y, Liu Q, Wang Y, Li G, Teng Q, Zhang Y, Liu S, Li Z (2015). Airborne transmission of a novel Tembusu virus in ducks. J Clin Microbiol.

[15] Zhang Y, Li X, Chen H, Ti J, Yang G, Zhang L, Lu Y, Diao Y (2015). Evidence of possible vertical transmission of Tembusu virus in ducks. Vet Microbiol.

[16] Su J, Li S, Hu X, Yu X, Wang Y, Liu P, Lu X, Zhang G, Hu X, Liu D, Li X, Su W, Lu H, Mok NS, Wang P, Wang M, Tian K, Gao GF (2011). Duck egg-drop syndrome caused by BYD virus, a new Tembusu-related flavivirus. PLoS One.

[17] Yan P, Zhao Y, Zhang X, Xu D, Dai X, Teng Q, Yan L, Zhou J, Ji X, Zhang S, Liu G, Zhou Y, Kawaoka Y, Tong G, Li Z (2011). An infectious disease of ducks caused by a newly emerged Tembusu virus strain in mainland China. Virology..

[18] Homonnay ZG, Kovacs EW, Banyai K, Albert M, Feher E, Mato T, Tatar-Kis T, Palya V (2014). Tembusu-like flavivirus (Perak virus) as the cause of neurological disease outbreaks in young Pekin ducks. Avian Pathol..

[19] Thontiravong A, Ninvilai P, Tunterak W, Nonthabenjawan N, Chaiyavong S, Angkabkingkaew K, Mungkundar C, Phuengpho W, Oraveerakul K, Amonsin A (2015). Tembusu-related Flavivirus in ducks, Thailand. Emerg Infect Dis.

[20] Yun T, Ye W, Ni Z, Zhang D, Zhang C (2012). Identification and molecular characterization of a novel flavivirus isolated from Pekin ducklings in China. Vet Microbiol.

[21] Li N, Lv C, Yue R, Shi Y, Wei L, Chai T, Liu S (2015). Effect of age on the pathogenesis of duck Tembusu virus in Cherry Valley ducks. Front Microbiol.

[22] Lu Y, Dou Y, Ti J, Wang A, Cheng B, Zhang X, Diao Y (2016). The effect of Tembusu virus infection in different week-old Cherry Valley breeding ducks. Vet Microbiol.

[23] Sun XY, Diao YX, Wang J, Liu X, Lu AL, Zhang L, Ge PP, Hao DM (2014). Tembusu virus infection in Cherry Valley ducks: the effect of age at infection. Vet Microbiol.

[24] Yan P, Li G, Wu X, Yan L, Teng Q, Li Z (2011). Rapid identification of duck Tembusu virus by the nested RT-PCR. Chin J Anim Infect Dis.

[25] Liu N, Jiang M, Wang M, Wang F, Zhang B, Zhang D (2016). Isolation and detection of duck astrovirus CPH: implications for epidemiology and pathogenicity. Avian Pathol.

[26] Wang F, Liang T, Liu N, Ning K, Yu K, Zhang D (2017). Genetic characterization of two novel megriviruses in geese. J Gen Virol.

[27] Liang T, Liu X, Cui S, Qu S, Wang D, Liu N, Wang F, Ning K, Zhang B, Zhang D (2016). Generation of a reliable full-length cDNA of infectious Tembusu virus using a PCR-based protocol. Virus Res.

[28] Pfeiffer J, Pantin-Jackwood M, Nguyen T, Suarez DL, To TL (2009). Phylogenetic and biological characterization of highly pathogenic H5N1 avian influenza viruses (Vietnam 2005) in chickens and ducks. Virus Res.

